# Supplementary material for: Deletions linked to PROG1 gene participate in plant architecture domestication in Asian and African rice
Source: Nat Commun. 2018 Oct 8;9:4157. doi: 10.1038/s41467-018-06509-2 (PMC6175861; doi:10.1038/s41467-018-06509-2)
Supplement: Supplementary file 1 — Supplementary Information [file 41467_2018_6509_MOESM1_ESM.pdf]

Deletions linked to *PROG1* gene participate in plant architecture domestication in Asian and African rice

Wu *et al*.

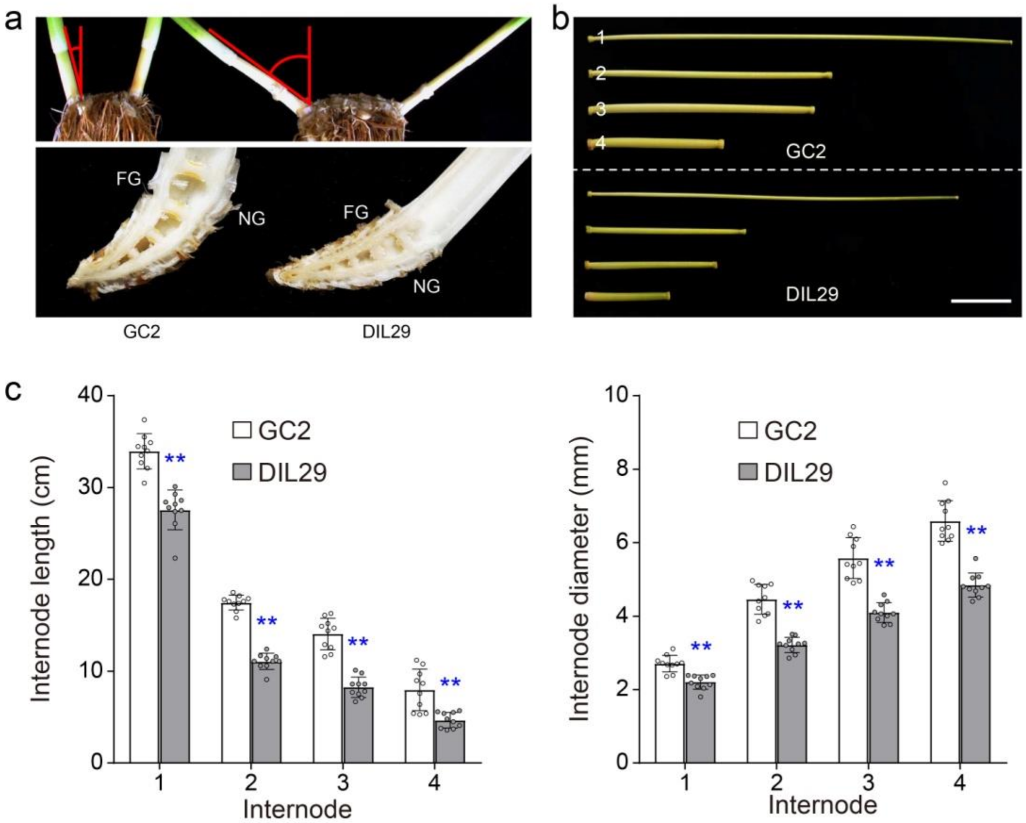

Supplementary Fig. 1 Characterization of the introgression line DIL29. **a** Comparison of the tiller base between Guichao 2 (GC2) and DIL29. FG, far ground; NG, near ground. **b** Comparison of the internodes in GC2 and DIL29. 1−4, top-one to top-four internodes. Scale bar, 5 cm. **c** The length and diameter measurements of the internodes in GC2 and DIL29. *X*-axis indicates the position of the internode counted from the top to base. Data are means (n = 10), with error bars showing standard deviation. **P < 0.01, Two-tailed Student's t-tests were performed.

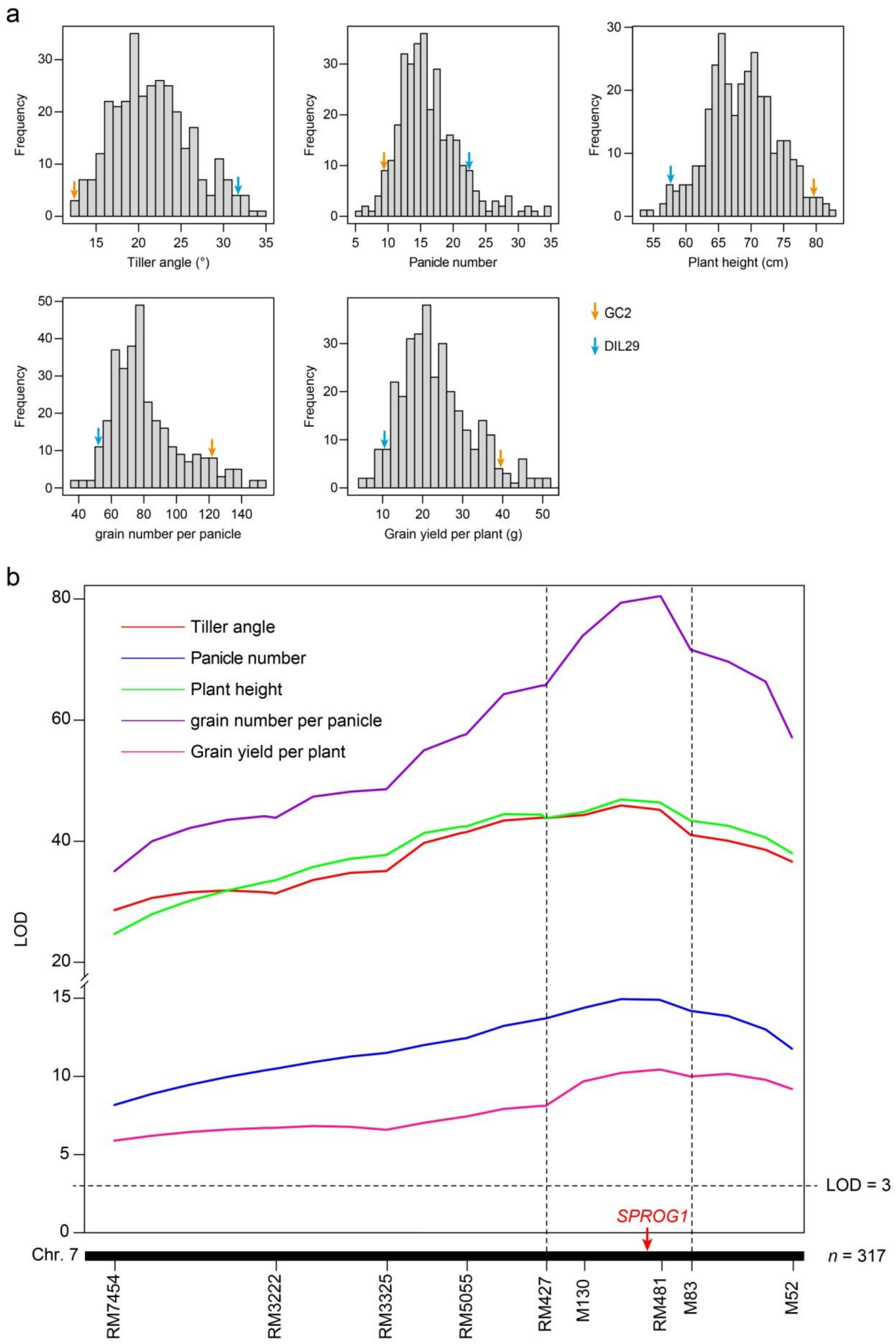

Supplementary Fig. 2 Mapping of the *SPROG1* locus. **a** The frequency distribution of five traits

(tiller angle, panicle number, plant height, grain number per panicle, and grain yield per plant) in the secondary F_2 population derived from the cross between Guichao 2 (GC2) and the introgression line DIL29. **b** QTL mapping of the five traits using the F_2 population. LOD, logarithm of odds.

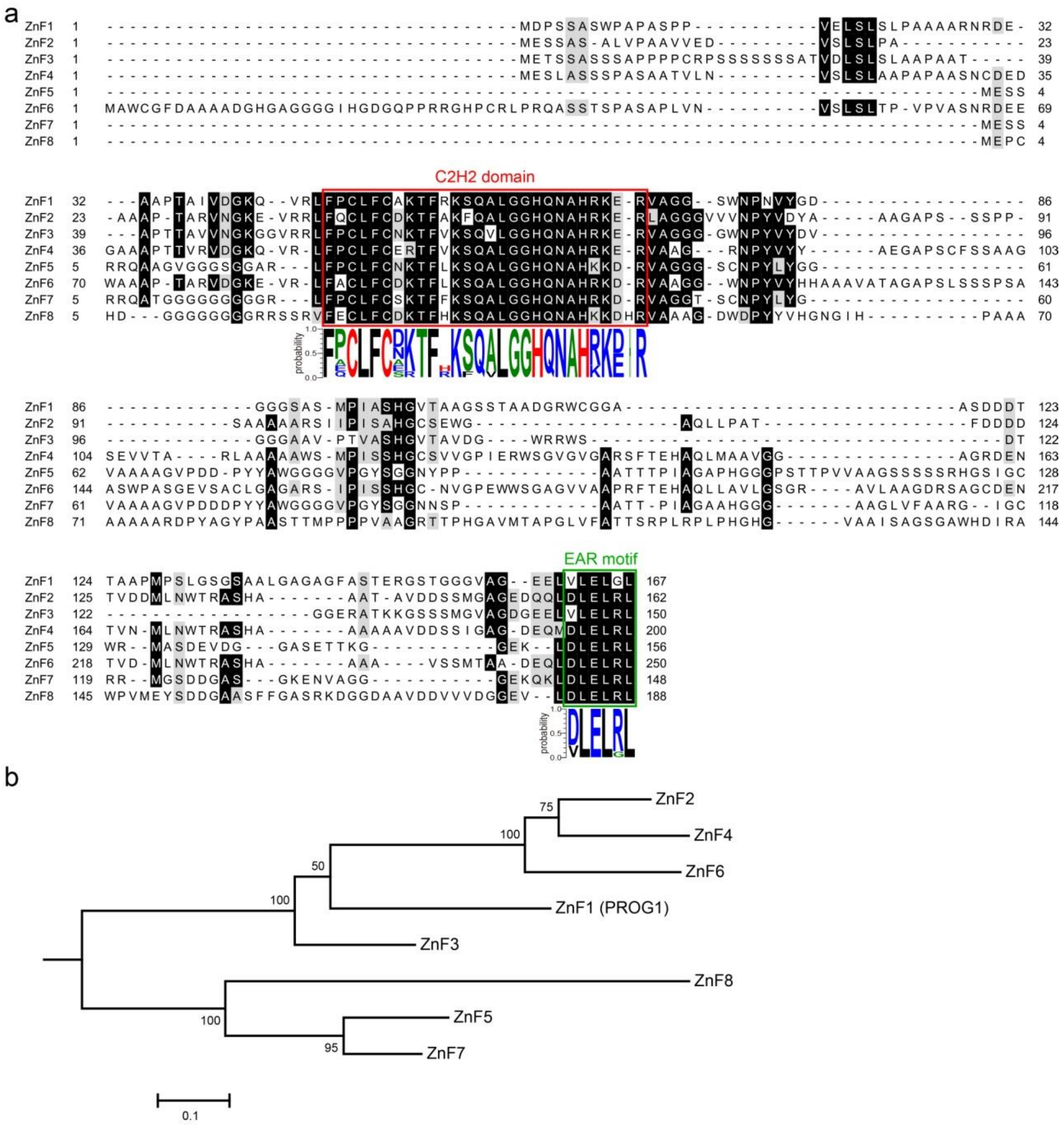

Supplementary Fig. 3 Amino acid sequence alignment (**a**) and phylogenetic analysis (**b**) of eight zinc-finger proteins at the *SPROG1* locus in *O. rufipogon* DXCWR. Multiple sequence alignment was performed using ClustalW. Black and grey boxes represent the positions of identical and similar sequences, respectively. Positions of the conserved Cys$_2$-His$_2$ (C2H2) zinc-finger domain and EAR motif are boxed in red and green, respectively. The logos graphically depict the sequence conservation of the Cys$_2$-His$_2$ (C2H2) zinc-finger domain, QALGGH domain and EAR motif. The neighbor-joining tree was constructed using MEGA 6.06 with 1,000 bootstrap replicates and the Jone-Taylor-Thornton (JTT) model.

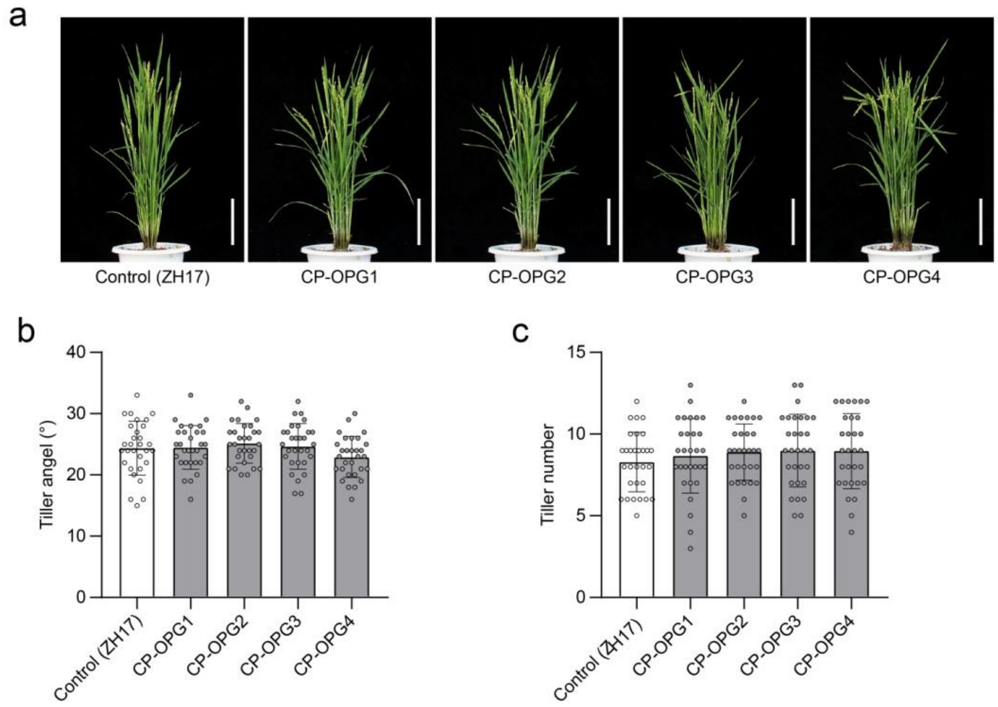

Supplementary Fig. 4 Complementary analyses of the other four constructs (OPG1 through OPG4). **a** Phenotypes of the transgenic plants (CP-OPG1 through CP-OPG4) and control plant (ZH17). Scale bars, 20 cm. **b**,**c** Comparison between the (**b**) tiller angle and (**c**) tiller number in the transgenic plants and control plant (ZH17). Data are means (n = 30), with error bars showing standard deviation. Two-tailed Student's t-tests were performed between ZH17 and transgenic plants (**$P < 0.01$).

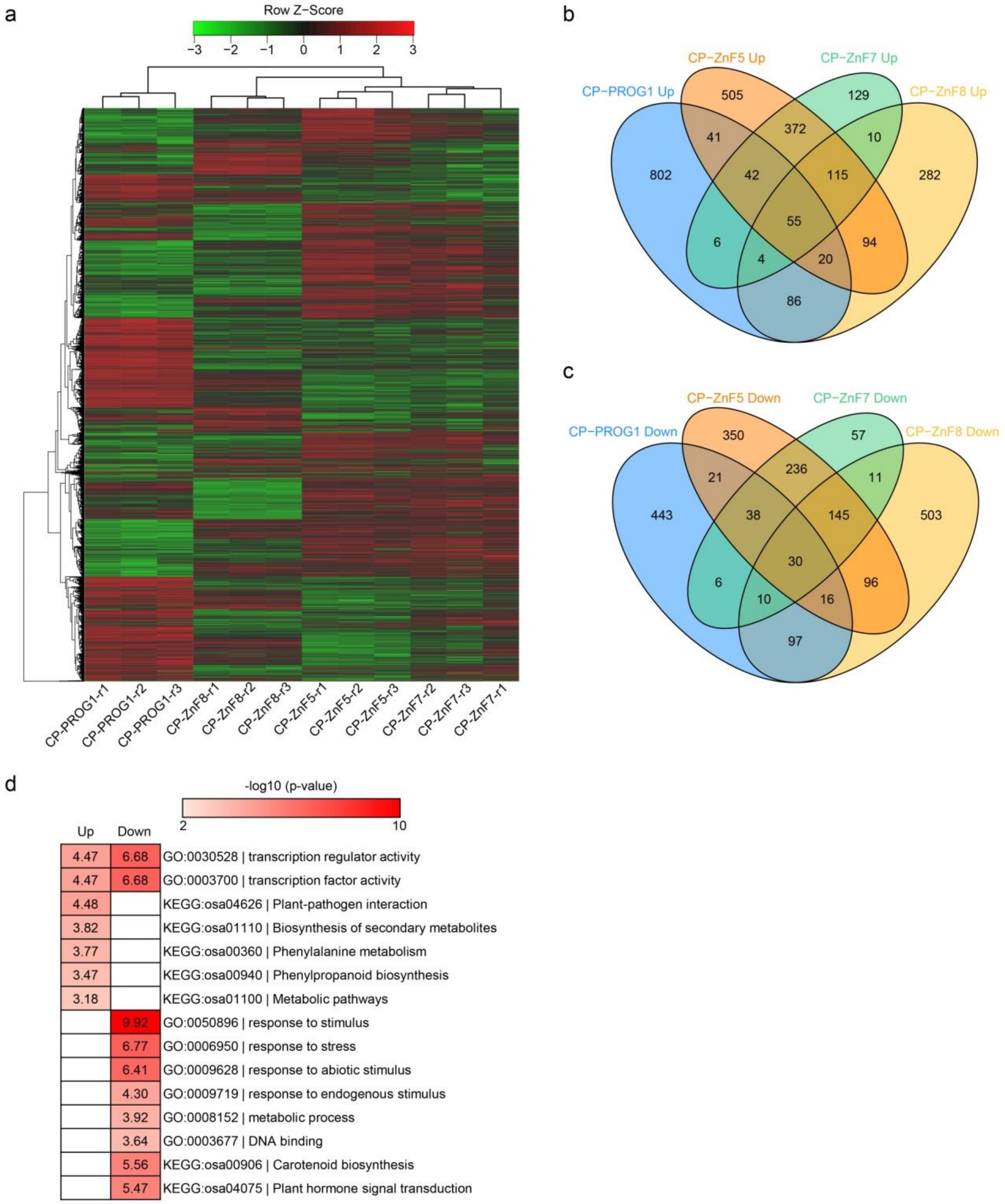

Supplementary Fig. 5 Transcriptome profiling via RNA-seq to investigate functions of four functional *ZnF* genes. **a** Hierarchical clustering of differentially expressed genes (DEGs). For each *ZnF* genes, all three biological replicates of RNA-seq are shown. **b** Venn diagrams analysis of up-regulated genes. **c** Venn diagrams analysis of down-regulated genes. **d** Gene ontology analysis of DEGs commonly regulated by both *ZnF5* and *ZnF7*.

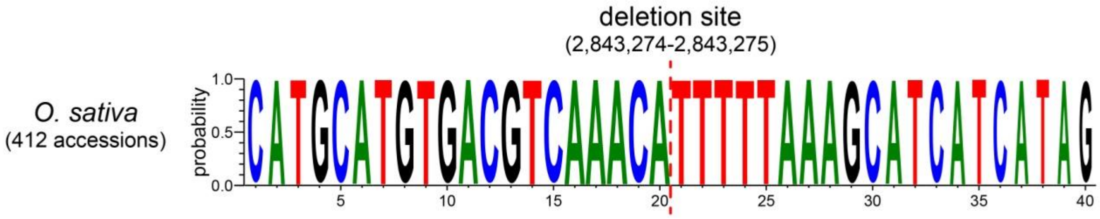

Supplementary Fig. 6 The consensus sequence of all reads covering the deletion site from 412 accessions. The breakpoint of deletion located between 2,843,274 bp to 2,843,275 bp on chromosome 7 in the Nipponbare reference genome (Os-Nipponbare-Reference-IRGSP-1.0, MSU7).

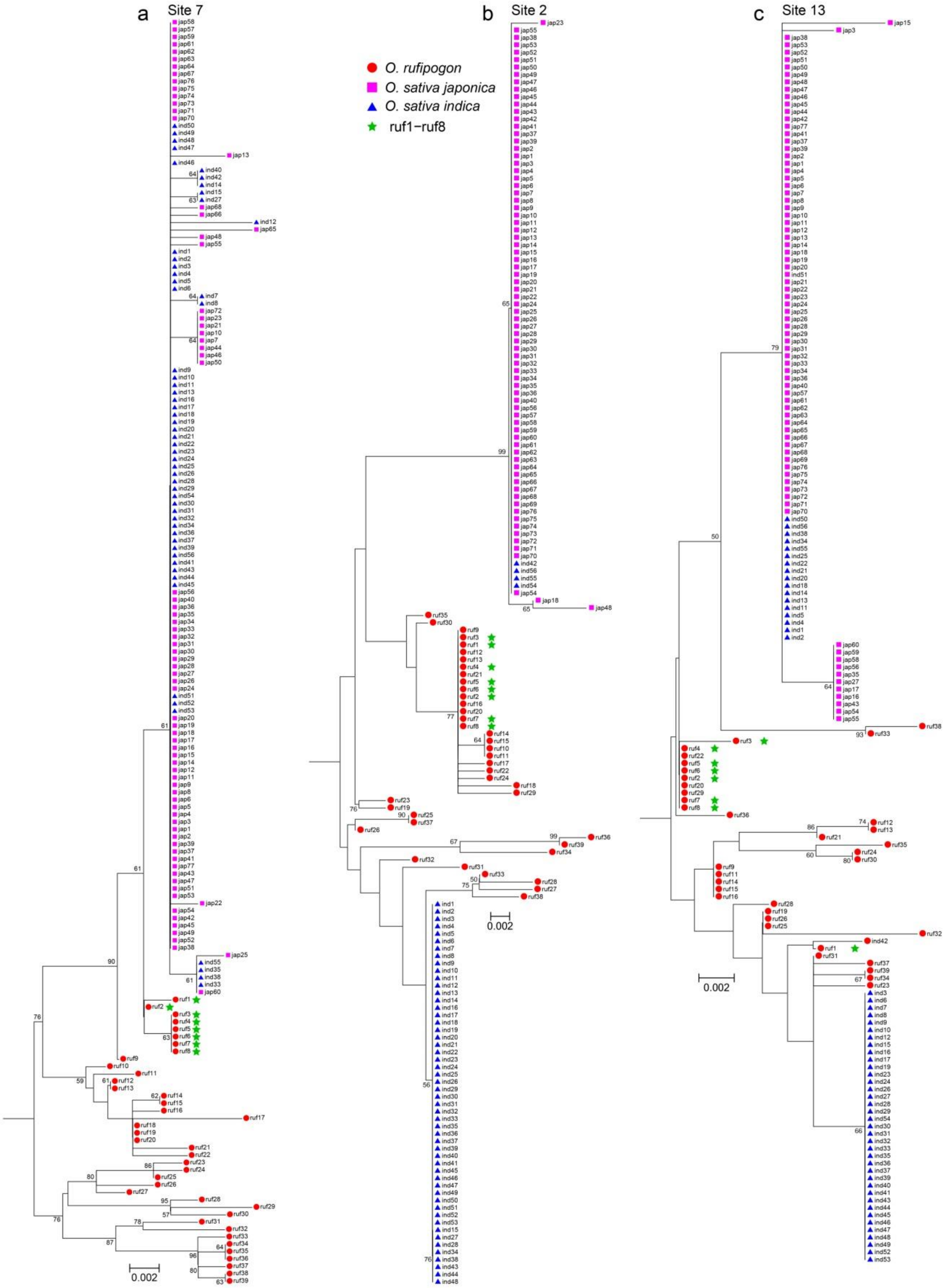

Supplementary Fig. 7 Phylogenies Neighbor-joining trees are shown at (**a**) Site 7, (**b**) Site 2 and (**c**) Site 13 from the sampled loci around the deletion site. The neighbor-joining tree was

constructed using MEGA 6.06 with 1,000 bootstrap replicates. The cut-off value for the consensus tree was 50%. The green asterisks denote the eight *O. rufipogon* accessions (ruf1−ruf8) that dispersed into this same clade of cultivated rice at Site 7. The *indica* (ind), *japonica* (jap) and *O. rufipogon* (ruf) samples used in this study are listed in Supplementary Data 4.

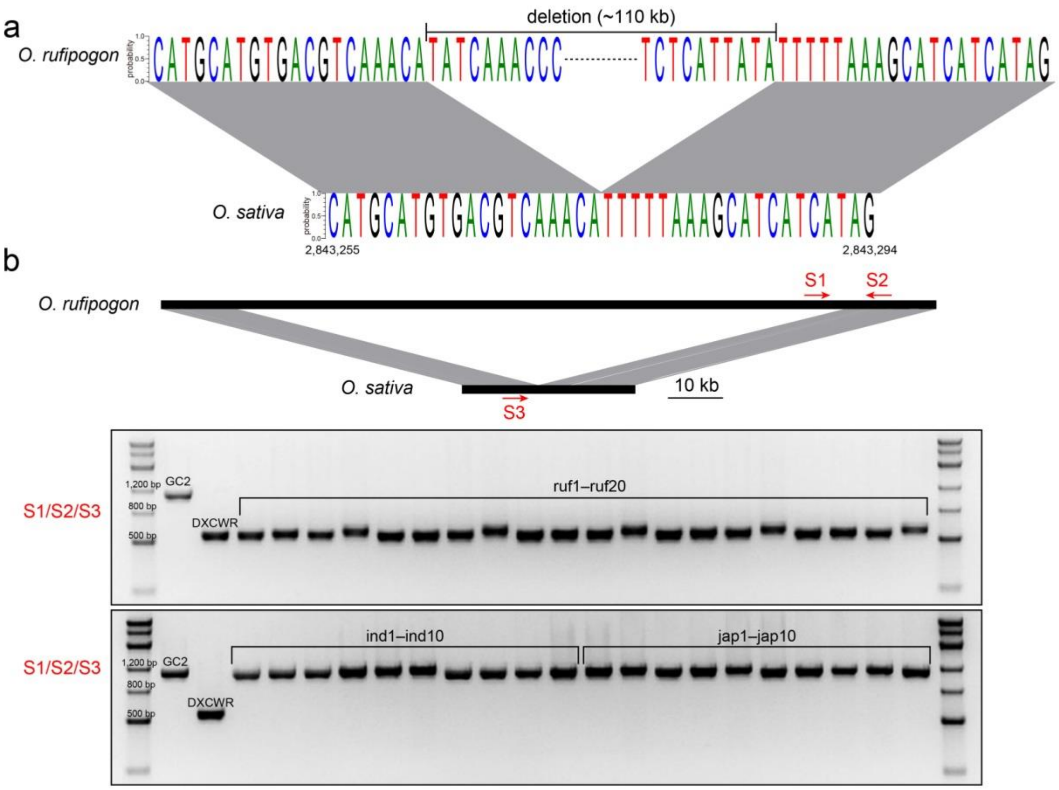

Supplementary Fig. 8 Identification of the deletion event in *O. rufipogon* and *O. sativa* genomes. **a** The sequences near the deletion site in *O. rufipogon* and *O. sativa* genome. **b** Identification of the deletion type in *O. rufipogon* and *O. sativa* by multiplex PCR. Red arrows show the location and direction of primers (S1-S3) used in multiplex PCR. The 10 *indica* (ind1−ind10), 10 *japonica* (jap1−jap10), and 20 *O. rufipogon* (ruf1−ruf20) samples are listed in Supplementary Data 4.

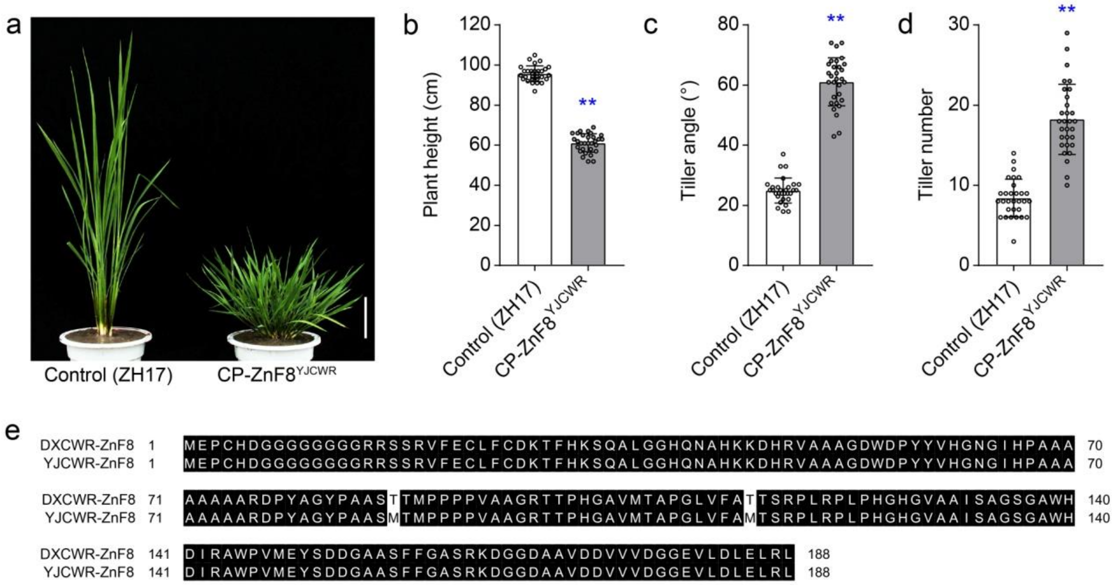

Supplementary Fig. 9 Phenotypes of CP-*ZnF8*^{YJCWR} transgenic plants. **a** Phenotypes of the CP-*ZnF8*^{YJCWR} transgenic plant and control plant (ZH17). Scale bars, 20 cm. **b**-**d** Comparison of the (**b**) plant height, (**c**) tiller angle and (**d**) tiller number in the control (ZH17) and CP-*ZnF8*^{YJCWR} transgenic plants. **e** Amino acid sequence comparison of ZnF8 in *O. rufipogon* YJCWR and ZnF8 in *O. rufipogon* DXCWR. Data are means (n = 30), with error bars showing standard deviation. Two-tailed Student's t-tests were performed between ZH17 and transgenic plants (**P < 0.01).

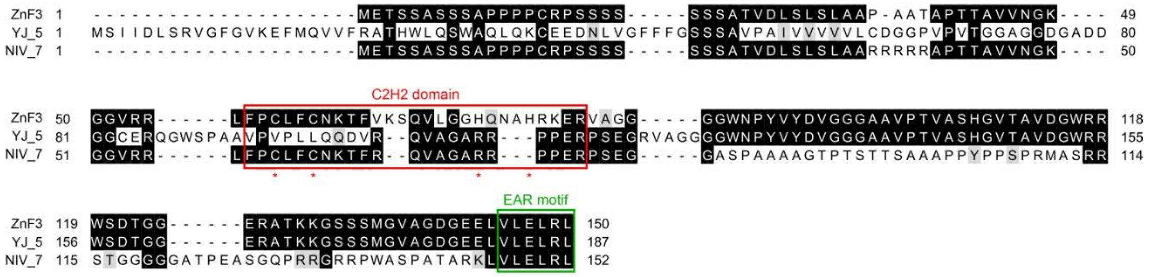

Supplementary Fig. 10 Amino acid sequence alignment of one zinc-finger protein within the *RPAD* locus among *O. rufipogon* DXCWR (ZnF3), *O. rufipogon* YJCWR (gene ID: YJ_5), and *O. nivara* W2014 (gene ID: NIV_7). Multiple sequence alignment was performed using ClustalW. Black and grey boxes represent the positions of identical and similar sequences, respectively. Positions of the conserved Cys_2-His_2 (C2H2) zinc-finger domain and EAR motif are boxed in red and green, respectively.

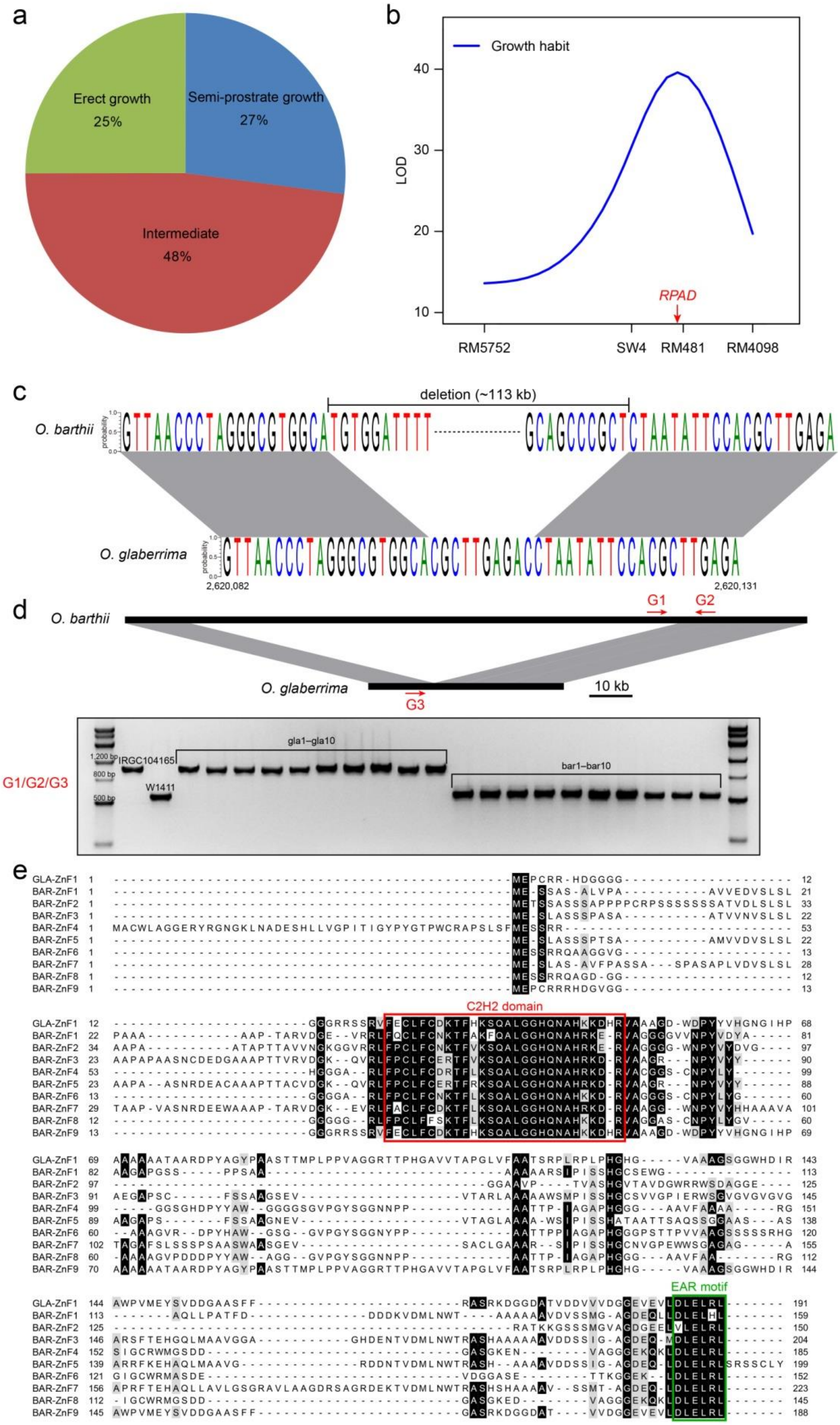

Supplementary Fig. 11 *RPAD* locus in African rice. **a** The frequency distribution of

growth habit trait in the F_2 population derived from the cross between *O. glaberrima* cultivar IRGC104165 (erect growth) and *O. barthii* accession W1411 (semi-prostrate growth). **b** A QTL associated with the transition of plant architecture between *O. barthii* and *O. glaberrima* was detected near the marker RM481, co-localizing with the *RPAD* locus. LOD, logarithm of odds. **c** The flanking genomic sequence covering the deletion site in 10 *O. barthii* accessions and 50 *O. glaberrima* varieties. All surveyed cultivars had an identical deletion. The numbers (2,620,082 and 2,620,131) showed the location of the 5′ and 3′ end in the *O. glaberrima* variety CG14 genome (http://ensembl.gramene.org/Oryza_glaberrima/, AGI1.1). **d** Identification of the deletion type in *O. barthii* and *O. glaberrima* using multiplex PCR. Red arrows show the location and direction of primers (G1−G3) used in multiplex PCR. The *O. glaberrima* (gla1−gla10) and *O. barthii* (bar1−bar10) samples are listed in Supplementary Data 4. **e** Amino acid sequence alignment of zinc-finger proteins at the *RPAD* locus in *O. glaberrima* (GLA-ZnF1) and *O. barthii* (BAR-ZnF1−BAR-ZnF9). Multiple sequence alignment was performed using ClustalW. Black and grey boxes represent the positions of identical and similar sequences, respectively. Positions of the conserved Cys_2-His_2 (C2H2) zinc-finger domain and EAR motif are boxed in red and green, respectively.

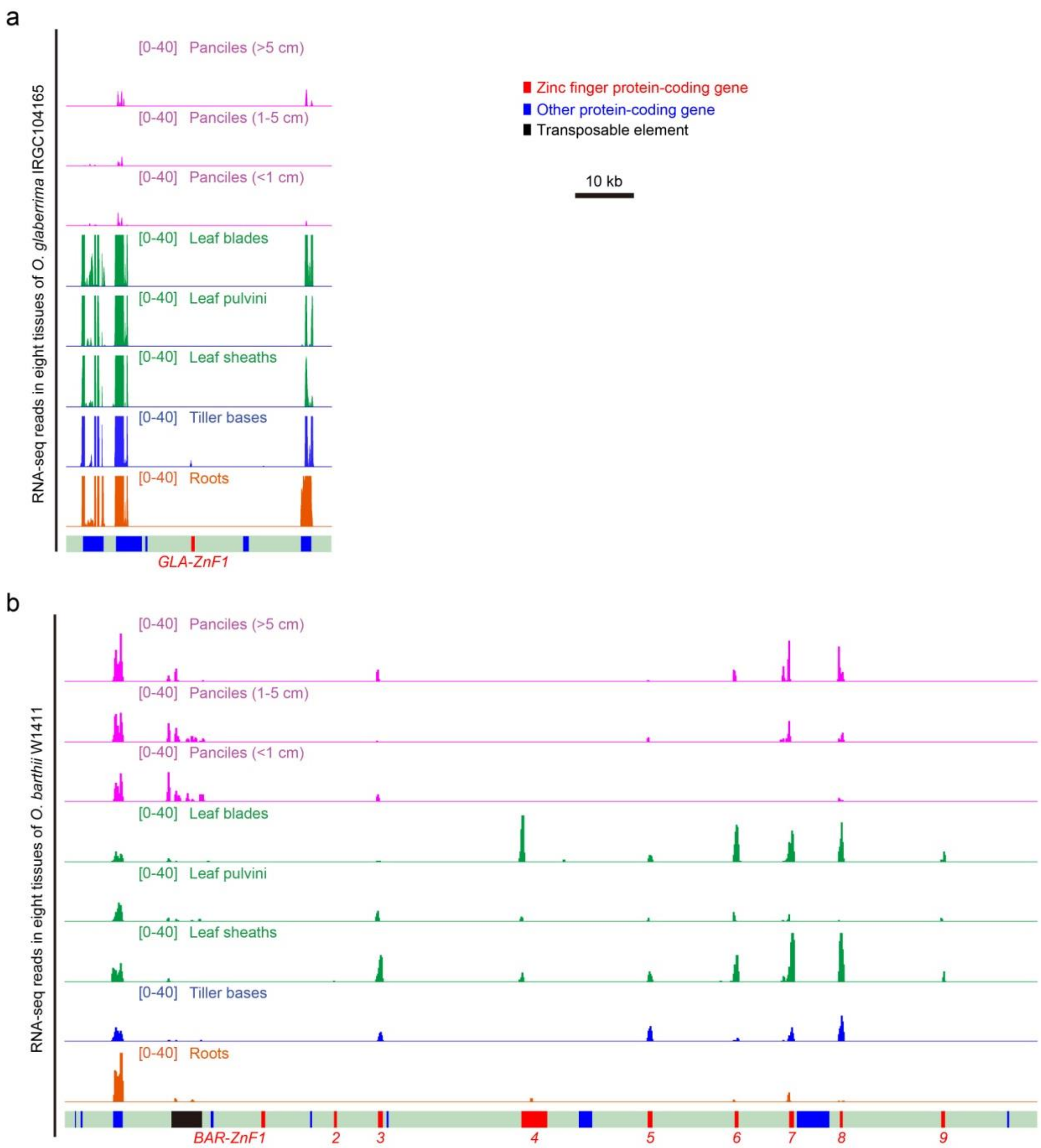

Supplementary Fig. 12 Expression profiles of genes within *RPAD* locus in *O. glaberrima* cultivar IRGC104165 (**a**) and *O. barthii* accession (**b**). The red, blue, and black boxes respectively represent zinc-finger protein-coding genes, other protein-coding genes, and transposable elements.

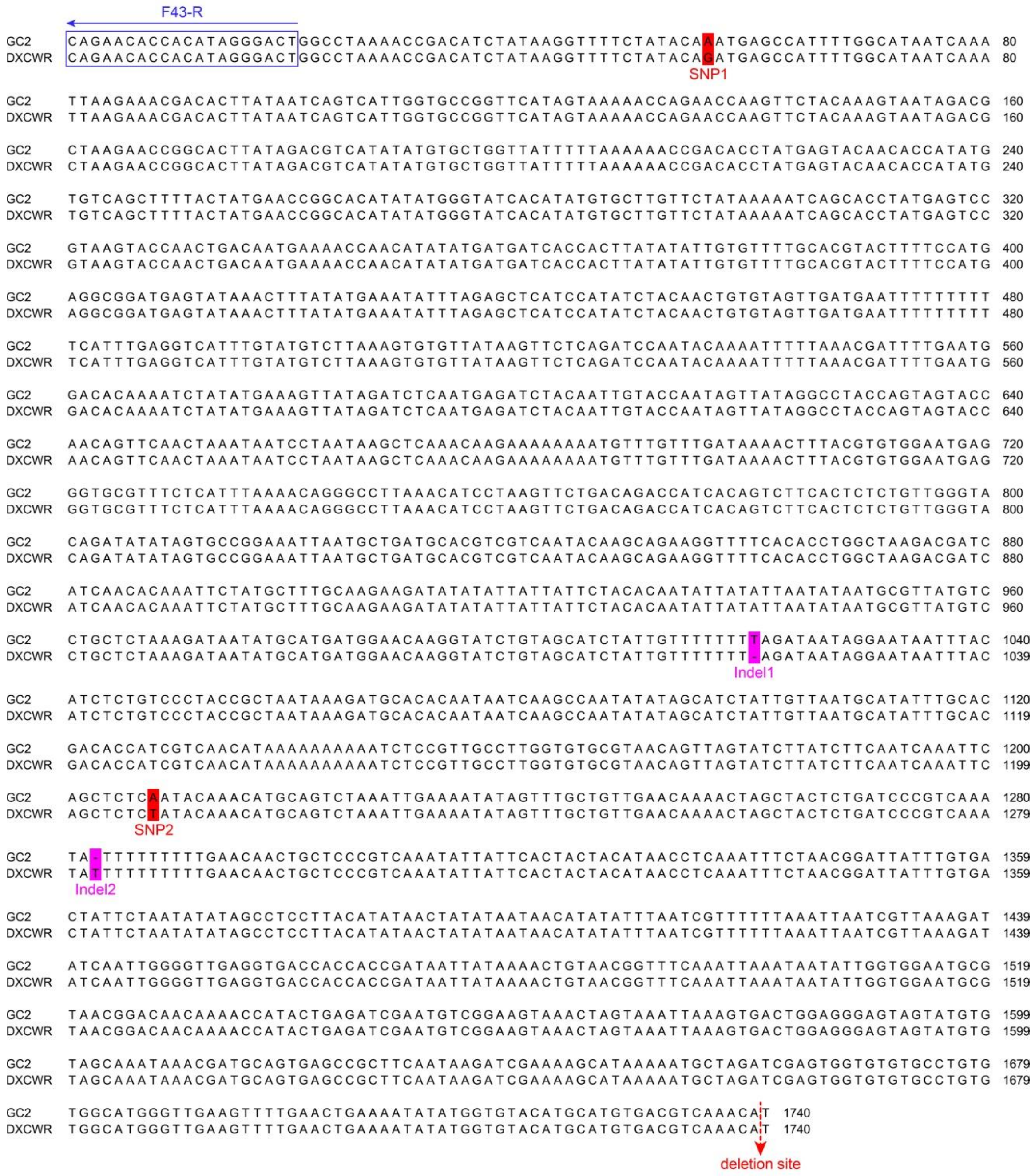

Supplementary Fig. 13 Comparison of 5′-flanking sequences of *PROG1* from the marker F43 to the deletion site between GC2 and DXCWR. Blue box represents the primer location of marker F43. The red vertical arrow indicates the deletion site.

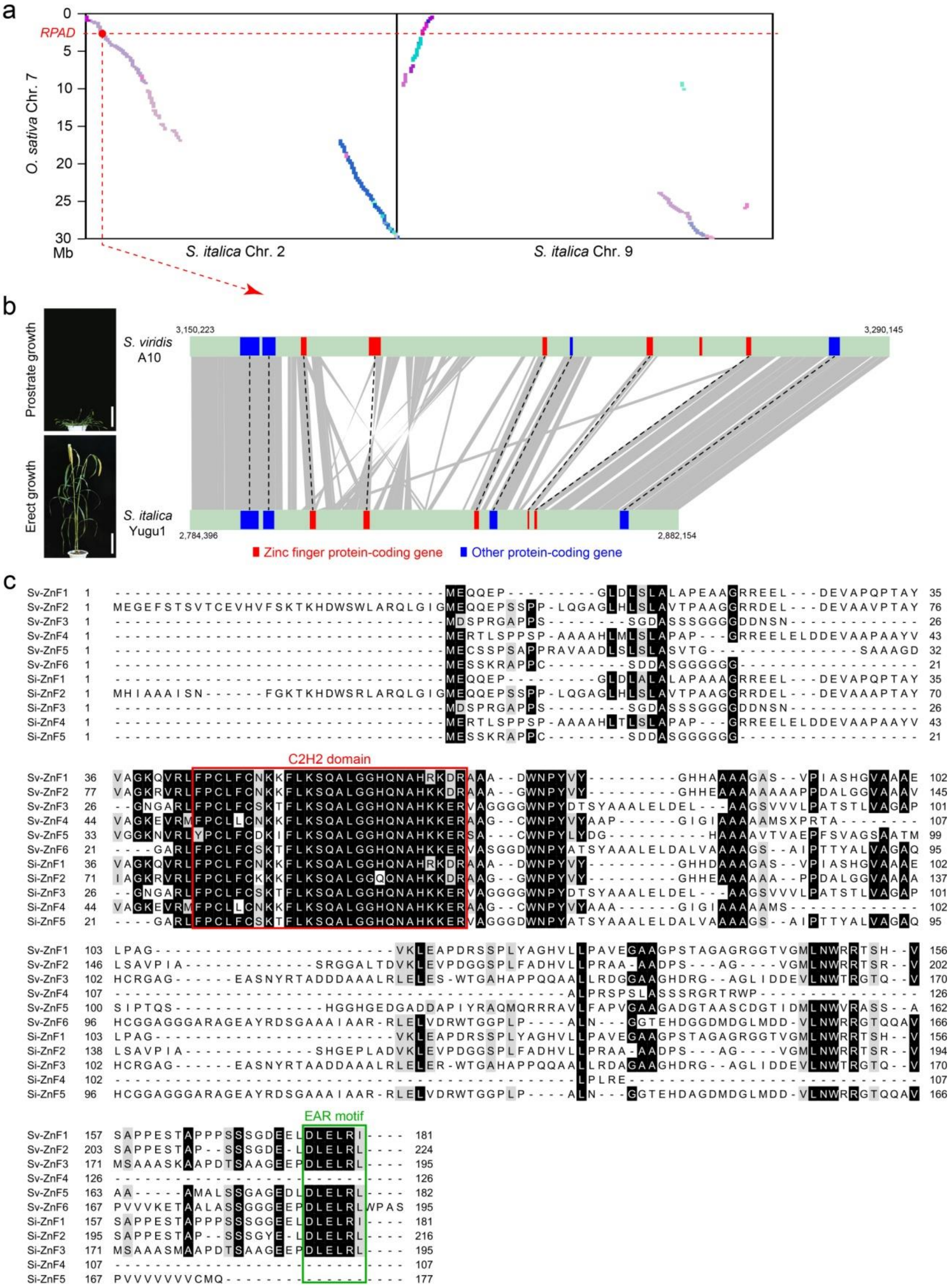

Supplementary Fig. 14 The *RPAD* syntenic region in *S. viridis* and *S. italica* genomes. **a** Genomic collinearity in *O. sativa* (variety Nipponbare) chromosome 7 and *S. italic* (variety Yugu 1) chromosome 2 (Chr. 2) and chromosome 9 (Chr.9). The genomic colinearity map was plotted

by MCScanX on the basis of the BLASTP. **b** Collinearity view of the *RPAD* locus in *S. viridis* accession A10 (v1.1, scaffold2: 3,150,223−3,290,145) and *S. italica* variety Yugu 1 (v2.2, Chr2: 2,784,396−2,882,154). Red and blue boxes indicate zinc-finger protein-coding genes and other protein-coding genes, respectively. The grey regions represent regions those sharing sequence collinearity. Dashed black lines indicate the orthologous relationships of genes. Scale bars, 20 cm. **c** Amino acid sequence alignment of zinc-finger proteins at the *RPAD* locus in *S. viridis* accession A10 (Sv-ZnF1−Sv-ZnF6) and *S. italica* variety Yugu 1 (Si-ZnF1−Si-ZnF5). Black and grey boxes represent the positions of identical and similar sequences, respectively. Positions of the conserved Cys_2-His_2 (C2H2) zinc-finger domain and EAR motif are boxed in red and green, respectively.

Supplementary Table 1 Comparison of the percentage of transposable elements in the whole reference genome of Nipponbare and deleted sequence of *O. rufipogon* DXCWR.

Transposable element	Percentage in Nipponbare reference genome (374,306,765 bp)	Percentage in deletion sequence of *O. rufipogon* DXCWR (110,041 bp)
Retroelements	22.61%	24.82%
SINEs	0.39%	0.53%
LINEs	0.88%	0.70%
L1/CIN4	0.88%	0.70%
LTR elements	21.34%	23.59%
Ty1/Copia	3.04%	0.77%
Gypsy/DIRS1	17.84%	17.62%
DNA transposons	14.18%	29.46%
hobo-Activator	0.57%	1.10%
Tc1-IS630-Pogo	2.42%	6.07%
Tourist/Harbinger	2.65%	4.89%
Unclassified	0.98%	0.46%
Total interspersed repeats	37.78%	54.74%

Supplementary Table 2 Description of the 11 complementary constructs in the *SPROG1* fine-mapping interval.

Constuct name	Corresponding gene	Size of insertion fragment (bp)	Method of obtaining fragment	Start[a]	End
CP-ZnF2	*ZnF2*	3,653	PCR amplification	16,399	20,051
CP-OPG1	*DX_4*	3,891	PCR amplification	25,095	28,985
CP-ZnF3	*ZnF3*	5,747	ultrasonication	27,082	32,828
CP-ZnF4	*ZnF4*	4,249	ultrasonication	33,141	37,389
CP-ZnF5	*ZnF5*	3,935	PCR amplification	41,701	45,635
CP-OPG2	*DX_9*	3,160	PCR amplification	55,953	59,112
CP-ZnF6	*ZnF6*	4,918	ultrasonication	60,511	65,428
CP-ZnF7	*ZnF7*	3,481	PCR amplification	71,867	75,347
CP-OPG3	*DX_16*	3,664	PCR amplification	89,718	93,381
CP-ZnF8	*ZnF8*	4,214	ultrasonication	116,649	120,862
CP-OPG4	*DX_20*	3,788	PCR amplification	126,481	130,268

a, The location in *O. rufipogon* DXCWR genome that is shown in **Fig. 2b**

Supplementary Table 3 Gene annotation of *RPAD* locus in *S. italica* variety Yugu1 and *S. viridis* accession A10.

Name	Gene ID[a]	*ZnF* gene[b]	Description	Length (bp)[c]	Start[d]	End	No. of exons	Strand
S. italica Yugu1	Seita.2G034600		DUP1365 protein	972	2,794,530	2,798,143	5	−
	Seita.2G034700		F-box kelch-repeat protein	1227	2,799,052	2,801,259	2	−
	Seita.2G034800	*Si-ZnF1*	C2H2 zinc finger protein	546	2,808,392	2,809,583	1	−
	Seita.2G034900	*Si-ZnF2*	C2H2 zinc finger protein	651	2,819,112	2,820,364	2	−
	Seita.2G035000	*Si-ZnF3*	C2H2 zinc finger protein	588	2,841,254	2,842,195	1	−
	Seita.2G035100		hypothetical protein	666	2,844,317	2,845,881	2	+
	Seita.2G035200	*Si-ZnF4*	C2H2 zinc finger protein	324	2,851,925	2,852,248	1	−
	Seita.2G035300	*Si-ZnF5*	C2H2 zinc finger protein	534	2,853,284	2,853,817	1	−
	Seita.2G035400		9-cis-epoxycarotenoid dioxygenase	1,749	2,870,377	2,872,125	1	+
S. viridis A10	Sevir.2G039200		DUP1365 protein	972	3,160,227	3,164,099	5	−
	Sevir.2G039300		F-box kelch-repeat protein	1227	3,164,689	3,167,317	2	−
	Sevir.2G039400	*Sv-ZnF1*	C2H2 zinc finger protein	546	3,172,394	3,173,512	2	−
	Sevir.2G039500	*Sv-ZnF2*	C2H2 zinc finger protein	675	3,185,987	3,188,363	3	+
	Sevir.2G039600	*Sv-ZnF3*	C2H2 zinc finger protein	588	3,220,691	3,221,600	1	−
	Sevir.2G039700		hypothetical protein	414	3,226,167	3,226,747	2	+
	Sevir.2G039800	*Sv-ZnF4*	C2H2 zinc finger protein	381	3,241,521	3,242,773	1	−
	Sevir.2G039900	*Sv-ZnF5*	C2H2 zinc finger protein	549	3,252,068	3,252,616	1	−
	Sevir.2G040000	*Sv-ZnF6*	C2H2 zinc finger protein	588	3,261,423	3,262,419	1	−
	Sevir.2G040100		9-cis-epoxycarotenoid dioxygenase	1749	3,277,956	3,280,131	1	+

a, The ID in the *S. italica* variety Yugu1 (v2.2) and *S. viridis* accession A10 (v1.1) genome (https://phytozome.jgi.doe.gov/pz/portal.html), respectively. b, Zinc finger protein-coding gene. c, Length of predicted ORF. d, For *S. italica* variety Yugu1 and *S. viridis* accession A10, the location was shown in their corresponding genomes (https://phytozome.jgi.doe.gov/pz/portal.html).
