# Supplementary material for: Deletions linked to PROG1 gene participate in plant architecture domestication in Asian and African rice
Source: Nat Commun. 2018 Oct 8;9:4157. doi: 10.1038/s41467-018-06509-2 (PMC6175861; doi:10.1038/s41467-018-06509-2)
Supplement: Supplementary file 3 — Description of Additional Supplementary Files [file 41467_2018_6509_MOESM3_ESM.pdf]

Descriptions of Additional Supplementary Files

File Name: Supplementary Data 1

Description: Gene annotation of the *RPAD* locus in the *indica* variety Guichao 2, *japonica* variety Nipponbare, *O. rufipogon* accession DXCWR, *O. rufipogon* accession YJCWR, and *O. nivara* accession W2014

File Name: Supplementary Data 2

Description: Differentially expressed genes detected in at least one *ZnF* transgenic plants

File Name: Supplementary Data 3

Description: Gene annotation of *RPAD* locus in *O. glaberrima* variety IRGC104165 and *O. barthii* accession W1411

File Name: Supplementary Data 4

Description: Plant materials used in this study

File Name: Supplementary Data 5

Description: Primers used in this study
